# Natural Language Processing for Work-Related Stress Detection Among Health Professionals: Protocol for a Scoping Review

**DOI:** 10.2196/56267

**Published:** 2024-05-15

**Authors:** Jannic Stefan Bieri, Catherine Ikae, Souhir Ben Souissi, Thomas Jörg Müller, Margarithe Charlotte Schlunegger, Christoph Golz

**Affiliations:** 1 Department of Health Professions Bern University of Applied Sciences Bern Switzerland; 2 School of Engineering and Computer Science Bern University of Applied Sciences Bern Switzerland; 3 Private Clinic Meiringen Bern Switzerland; 4 Translational Research Center University Hospital of Psychiatry and Psychotherapy University of Bern Bern Switzerland

**Keywords:** health professionals, natural language processing, text-mining, work-related stress, healthcare, occupational well-being, automatic detection, scoping review protocol, methodology, synthesis

## Abstract

**Background:**

There is an urgent need worldwide for qualified health professionals. High attrition rates among health professionals, combined with a predicted rise in life expectancy, further emphasize the need for additional health professionals. Work-related stress is a major concern among health professionals, affecting both the well-being of health professionals and the quality of patient care.

**Objective:**

This scoping review aims to identify processes and methods for the automatic detection of work-related stress among health professionals using natural language processing (NLP) and text mining techniques.

**Methods:**

This review follows Joanna Briggs Institute Methodology and PRISMA-ScR (Preferred Reporting Items for Systematic Reviews and Meta-Analyses Extension for Scoping Reviews) guidelines. The inclusion criteria for this scoping review encompass studies involving health professionals using NLP for work-related stress detection while excluding studies involving other professions or children. The review focuses on various aspects, including NLP applications for stress detection, criteria for stress identification, technical aspects of NLP, and implications of stress detection through NLP. Studies within health care settings using diverse NLP techniques are considered, including experimental and observational designs, aiming to provide a comprehensive understanding of NLP’s role in detecting stress among health professionals. Studies published in English, German, or French from 2013 to present will be considered. The databases to be searched include MEDLINE (via PubMed), CINAHL, PubMed, Cochrane, ACM Digital Library, and IEEE Xplore. Sources of unpublished studies and gray literature to be searched will include ProQuest Dissertations & Theses and OpenGrey. Two reviewers will independently retrieve full-text studies and extract data. The collected data will be organized in tables, graphs, and a qualitative narrative summary. This review will use tables and graphs to present data on studies’ distribution by year, country, activity field, and research methods. Results synthesis involves identifying, grouping, and categorizing. The final scoping review will include a narrative written report detailing the search and study selection process, a visual representation using a PRISMA-ScR flow diagram, and a discussion of implications for practice and research.

**Results:**

We anticipate the outcomes will be presented in a systematic scoping review by June 2024.

**Conclusions:**

This review fills a literature gap by identifying automated work-related stress detection among health professionals using NLP and text mining, providing insights on an innovative approach, and identifying research needs for further systematic reviews. Despite promising outcomes, acknowledging limitations in the reviewed studies, including methodological constraints, sample biases, and potential oversight, is crucial to refining methodologies and advancing automatic stress detection among health professionals.

**International Registered Report Identifier (IRRID):**

PRR1-10.2196/56267

## Introduction

Worldwide health care systems are in urgent need of qualified health professionals. The global demand–driven shortage of health professionals is expected to exceed 14 million by the year 2030 [1]. There are many reasons for the shortage, such as the aging of society, the increase in medical and technological treatment options, and the high workload on health care staff [2-4]. The pandemic has put additional strain on an already tense situation. During the initial phase of the pandemic in March 2020, health professional shortages increased by 62% [5], and over 80% of nurses experienced psychological distress [6]. The already high contemplation of quitting one’s job increased due to the pandemic by up to 20.3% [7].

During the COVID-19 pandemic, health professionals have faced increased pressure, including heavy workloads, job-related stress, physical and mental health risks, and even instances of violence and harassment [8]. Furthermore, high stress levels and understaffing at the workplace are associated with negative impacts on patient safety and the quality of care [9]. Workplace dissatisfaction and psychological stress significantly increase the risk of mental health issues, such as burnout [10]. As a result, many health professionals leave the profession within the first 5 years after graduation [10]. To reduce the turnover rate of health professionals, it is crucial to reduce workplace stressors and improve working conditions [9]. However, the implementation of international or national measures requires adequate monitoring of the situation, especially regarding working conditions [1].

In this context, the early detection of work-related stress becomes particularly relevant. Previous research in this area has primarily relied on studies that collected self-reported data from health professionals through surveys [11]. However, the response rate in these study designs is decreasing and a further decline is expected [12]. Conducting instant monitoring is time-consuming, which underscores the benefits of automatic stress detection [13].

In recent decades, there has been continuous innovative development in the field of information technology, with these milestones contributing to the advancement of e-health care in terms of quality, continuity, and efficiency [14]. An example of this progress is the use of sensors that can capture physiological data, including the use of smartphones and wearable sensors, which allow the detection of biomarkers to measure stress. These sensors not only have the capacity to identify stress but also accurately categorize and differentiate various emotions [15]. A systematic review examined the use of wrist-worn wearables for the identification of stress levels. The results show that the development of such devices with physiological and chemical sensors is possible but challenging [16]. Another systematic review aimed to investigate and summarize the literature on physiological responses to acute stress, revealing a focus on retrospective assessment, with a notable gap in methodological approaches suitable for health care. The findings emphasize the need for future research to leverage sensor technology for real-time evaluation of acute stress in health professionals [17].

A promising application area that has made significant progress involves the use of natural language processing (NLP) and text mining techniques for recognizing clinical information in unstructured free-text documents and transforming it into structured data [18]. NLP, a technique within the broader field of artificial intelligence, has rapidly evolved in the past 2 decades with the increasing proliferation of computer technology. NLP is now used daily by every smartphone user and has significantly contributed to the development of speech translation, personal digital assistants, and voice-controlled home automation systems [19]. The primary goal of NLP is to view text or speech input or output not only as a sequence of characters, sentences, phrases, or paragraphs but rather as a complex syntactic and phonological data structure [20].

NLP is an algorithmic tool that identifies and manages texts, similar to text mining. Text mining involves various techniques for characterizing and converting texts. Within text mining, NLP methods are applied based on syntactic or semantic processing algorithms to analyze text data [21]. Text mining is a part of NLP. We will use the term “NLP,” which includes text mining. NLP techniques have been applied in various health care areas for decision-making processes, including mental health and oncology [22,23]. One study investigated the use of NLP to analyze social media posts for inferring mental states and creating personalized mental health interventions, fostering a connection between mental health, human-computer interaction, and NLP fields [22]. Another study used NLP as a tool for enhancing evidence-based oncological research and improving cancer care quality [23].

NLP was also used for stress detection among patients. A study examines the automated assessment of patient self-narratives for signs of posttraumatic stress disorder (PTSD) using NLP [24]. The authors use NLP techniques to identify patterns in patients’ texts. The key results show that automated analysis of patient texts holds promise in recognizing potential indicators of PTSD. This approach could be efficient for the early detection and diagnosis of PTSD, especially in large volumes of patient narratives [24]. NLP could also be used as an opportunity to transform mental health conversation and help early intervention for depression [25]. It is indicated to detect stress and mental disorders in an early stage [25]. However, the use of NLP extends not only to the detection of stress in patients but also to the field of health professionals. Under these promising conditions, the extent to which NLP can be used to identify workplace stress among health professionals needs to be clarified.

The main goal of this scoping review is to identify processes and methods for the automatic detection of work-related stress among health professionals using NLP and text mining. By examining these innovative approaches, the authors aim to contribute to the improvement of health and well-being among health professionals.

The review question includes the following: Which processes and methods for automatic work-related stress detection using NLP among health professionals have been identified in the scientific literature?

## Methods

### Overview

This scoping review has not been registered on any public platform. A preliminary search was conducted in the PROSPERO, MEDLINE, and Cochrane databases for systematic reviews and Joanna Briggs Institute Evidence Synthesis. No existing or ongoing scoping or systematic review on this topic could be identified. The proposed scoping review will follow the Joanna Briggs Institute methodology for scoping reviews [26]. The PRISMA-ScR (Preferred Reporting Items for Systematic Reviews and Meta-Analyses Extension for Scoping Reviews) checklist will also be applied [27].

### Participants

This scoping review will consider studies that include health professionals and focus on automatically detecting work-related stress using NLP. Other professions and children are excluded.

### Concept

This scoping review examines the detection of work-related stress among health professionals through NLP. The investigation covers different aspects related to this topic. Initially, it focuses on the scope of NLP applications for stress detection. This involves using NLP methods and techniques to analyze text data produced by health professionals to identify signs of work-related stress.

Another crucial aspect involves the features and criteria for stress detection, encompassing the definition of text features and criteria that could indicate the presence of stress. These may include specific linguistic patterns, keywords, or other linguistic indicators.

Within the scope of this review, the technical aspects of NLP approaches are also examined, encompassing the various NLP methods and techniques that can be used for stress detection in health professionals. These methods include machine learning, sentiment analysis, text mining, and data mining.

Last, the review illuminates the results and implications of this automatic stress detection through NLP, including potential enhancements in identifying stressed health professionals, facilitating the implementation of stress management measures, promoting a better understanding of stress factors in health care, and the potential improvement in patient care through efficient stress management.

These concepts serve as a foundation for the identification of relevant studies and research works that deal with the automatic detection of work-related stress among health professionals using NLP techniques.

### Context

This review will encompass studies focusing on the application of NLP in the health care domain. It will encompass investigations conducted in diverse environments, including hospitals, clinics, and health care facilities, where NLP techniques are used to automatically detect work-related stress among health professionals. Emphasis will be placed on the use of textual data generated by health professionals to identify stress patterns and enhance the mental health and well-being of the workforce. This context extends across various health care sectors and settings, where the implementation of NLP for stress detection holds the potential for positive impacts on working conditions and patient care.

### Types of Sources

This review paper considers various types of study designs in the field of NLP. This includes experimental studies such as randomized controlled trials, cluster-randomized trials, pre- and posttest designs, and qualitative studies that investigate the effects of NLP techniques on stress detection in health professionals. Analytical observational studies, including prospective and retrospective cohort studies as well as case-control studies, are also included to analyze potential associations between the use of NLP and stress among health professionals. Descriptive observational studies, such as case series, individual case reports, and descriptive cross-sectional studies, provide insights into the application of NLP for stress detection in real health care settings. Furthermore, observational studies are considered to monitor the behavior of health professionals in dealing with NLP systems and document their impact on stress. Last, expert opinions and evaluations from professionals in the field of NLP and health care are incorporated to obtain best practices and recommendations. The inclusion of these various types of sources allows for a comprehensive understanding of the application of NLP for stress detection in health professionals and to gain relevant insights from different perspectives.

### Search Strategy

The search strategy was developed to capture both published and unpublished studies. This process commenced with defining the objectives and establishing inclusion and exclusion criteria. Subsequently, the PICO (population, intervention, control, and outcomes) framework was constructed, suitable databases were identified, and search components and keywords were defined. Initially, a preliminary limited search was conducted in PubMed and CINAHL (via EBSCOhost) to gain an overview of the available results. Based on these findings, the search strategy was further refined.

A second search will be conducted using the adapted keywords across all relevant databases. A detailed search strategy conducted on October 5, 2023, for PubMed is extensively described in Table 1. The reference list of all included sources of evidence will be screened for additional studies.

Papers in English, German, and French were included to encompass a broad range of literature sources. Studies published from 2013 to the present were included to ensure that current literature is integrated into the analysis. This timeframe was justified by a significant increase in the number of publications, particularly after 2012 [28].

**Table 1:** Preliminary search on PubMed.

Search number	Query	Records retrieved
1	(((((((((caregiver) OR (nurse)) OR (allied health personnel)) OR (physician)) OR (health personnel)) OR (medical professional)) OR (clinician)) OR (medical practitioner)) OR (health providers)) OR (health professional)	2,930,096
2	((NLP^a^) OR (natural language processing)) OR (text mining)	51,668
3	((((((((((work-related stress) OR (job-related stress)) OR (occupational stress)) OR (workplace stress)) OR (work stress)) OR (job stress)) OR (occupational pressure)) OR (work-induced stress)) OR (professional stress)) OR (work-related strain)) OR (job-related tension)	165,798
4	#1 AND #2 AND #3	70
5	Limited to English, German, French, and abstract available	68

^a^NLP: natural language processing.

### Information Sources

The databases to be searched include MEDLINE (via PubMed), CINAHL (EBSCOhost), PubMed, Cochrane, ACM Digital Library, and IEEE Xplore. In addition to the mentioned databases, sources for unpublished studies and gray literature will also be searched, including ProQuest Dissertations & Theses and OpenGrey. This comprehensive search strategy was developed to ensure the inclusion of a wide range of relevant studies and information for the research.

### Study Selection and Extraction

Following the search, all identified citations will be collated and uploaded into Zotero (version 6.0.27; Roy Rosenzweig Center for History and New Media), and the duplicates will be removed. Titles and abstracts are then screened for assessment by 2 independent reviewers, following the inclusion criteria. Two reviewers will independently extract data from each eligible paper. Papers are retrieved and evaluated in detail based on the inclusion criteria. Full texts that do not meet the inclusion criteria are excluded, and the reasons for exclusion are provided in an appendix in the review’s final report. Any disagreements that arise between the reviewers at each stage of the selection process will be resolved through discussion, or with an additional reviewer or reviewers. The results of the search and the study inclusion process will be reported in full in the final scoping review and presented in a PRISMA-ScR flow diagram [27].

Data will be extracted from papers included in the scoping review by 2 or more independent reviewers using a data extraction tool developed by the reviewers. The descriptive data extracted will include specific details about the participants, concept, context, study methods, and key findings relevant to the review question, along with the evidence levels [29] of the respective studies. A draft of the data collection instrument is provided in Textbox 1. It is modified and revised from each included paper during the data collection process. Modifications are detailed as part of the full review. Authors of the papers are contacted to request for missing or additional data if necessary.

**The data to be extracted.**

- Authors
- Year
- Title
- Countries
- Context or setting
- Participants
- Purpose or objective
- Research methods
- Natural language processing methods and techniques for stress detection
- Features and criteria for stress detection
- Evidence level

### Data Analysis

The extracted data will be presented in tables or graphs, depending on the objectives and scope of this review. The tables and graphs will report on the distribution of studies by publication year or timeframe, countries of origin, fields of activity, and research methods used. The research methods used will be extracted to depict the range of available evidence, including both quantitative and qualitative aspects on the subject. Descriptive qualitative data synthesis of text data from the studies involves the following 3 steps: identifying results, grouping results, and categorizing groups. A narrative summary accompanies the tables or graphically represented results using a PRISMA-ScR flow diagram and describes how the findings relate to the review’s objectives and questions. The results are discussed in terms of their implications for practice and research.

## Results

We anticipate that the final outcomes will be presented in a systematic scoping review by June 2024.

## Discussion

### Principal Findings

The main findings of this scoping review will present several key aspects regarding the automatic detection of work-related stress among health professionals using NLP and text mining techniques. This scoping review could successfully identify a range of processes and methods for the automatic detection of work-related stress among health professionals using NLP and text mining techniques. Analysis of the literature will reveal various approaches used in detecting stress, including machine learning algorithms, sentiment analysis, and text mining methodologies. The findings will underscore the potential of NLP and text mining in revolutionizing stress detection methods within health care settings. By improving advanced technological tools, health care institutions could potentially integrate these approaches into their systems to streamline stress monitoring processes and enhance the well-being of health professionals. The discussion further clarifies the implications of these findings for both practice and research domains, emphasizing the importance of adopting innovative strategies to moderate workplace stress among health professionals.

### Comparison With Prior Work

Comparisons to existing literature highlight the novelty and significance of using NLP and text mining for stress detection in health professionals. Traditionally, stress detection relied on self-reported data, often gathered through surveys, resulting in diminishing response rates [11,12]. Immediate monitoring proves to be time-consuming, underscoring the advantages of automated stress detection [13]. This scoping review demonstrates the feasibility and benefits of automated stress detection methods. Furthermore, the scoping review explores how these findings align with and contribute to the broader field of literature on stress management and occupational health within health care settings.

### Limitations

Despite the promising outcomes, it is crucial to recognize the limitations present in the reviewed studies. These limitations may include methodological constraints, sample biases, and technological challenges associated with NLP and text mining applications. While the authors attempt to search for unpublished studies and gray literature, it is essential to acknowledge the possibility of oversight, which could introduce bias to the authors’ comprehension and conclusions. Furthermore, limiting our scope to studies from the past decade may lead to a time bias, potentially excluding relevant information from earlier literature. However, concerning the recent advances in NLP in the last years and an exponential increase in publications on that topic, we expect to identify the most relevant publications. Additionally, including only English, French, and German studies may introduce a language bias, potentially overlooking relevant research published in other languages. Future research can refine methodologies and advance automatic stress detection among health professionals by addressing these limitations.

### Conclusions

This scoping review fills a significant gap in the existing literature by identifying automated methods for detecting work-related stress among health professionals using NLP and text mining. The study’s outcomes may offer valuable insights into the potential applications of these technologies in health care settings, emphasizing the importance of proactive stress management strategies. Moreover, the review highlights the potential for future research and underscores the need for ongoing investigation into the efficacy and implementation of NLP-based interventions.

## Abbreviations

NLP: natural language processing
PRISMA-ScR: Preferred Reporting Items for Systematic Reviews and Meta-Analyses Extension for Scoping Reviews
PICO: population, intervention, control, and outcomes
PTSD: posttraumatic stress disorder

## Data Availability

All data generated or analyzed during this study are included in the final scoping review publication and its supplementary information files.
